# The Effect of In Ovo Administration of Rosemary Essential Oil on Hatchability, Relative Hatching Weight, and Embryo Mortality Rate in Japanese Quail (*Coturnix coturnix japonica*)

**DOI:** 10.3390/ani13071217

**Published:** 2023-03-31

**Authors:** Assia Aberbour, Leghel Touazi, Amine Benberkane, Sofiane Aissanou, Anjum Sherasiya, Mokrane Iguer-Ouada, Jean Luc Hornick, Nassim Moula

**Affiliations:** 1 Department of Veterinary Management of Animal Resources, Faculty of Veterinary Medicine, University of Liege, 4000 Liege, Belgium; 2 Department of Agronomy, Faculty of Nature and Life Sciences, Ferhat Abbas University of Setif, EL Bez, Setif 19000, Algeria; 3 Associated Laboratory in Marine Ecosystems and Aquaculture, Department of Biological Sciences of the Environment, Faculty of Nature and Life Sciences, University of Bejaia, Bejaia 06000, Algeria; 4 Veterinary World, Star, Gulshan Park, NH-8A, Chandrapur Road, Wankaner 363621, India; 5 Fundamental and Applied Research for Animal and Health (FARAH), University of Liege, 4000 Liege, Belgium; 6 GIGA-Animal Facility, University of Liege, 4000 Liege, Belgium

**Keywords:** in ovo, essential oil, Japanese quail (*Coturnix coturnix japonica*), rosemary, *Rosemarinus officinalis*, hatching parameters

## Abstract

**Simple Summary:**

In poultry, supplementing diets with essential oils has gained much interest. It has been established that supplementing avian diets and water with rosemary essential oil improves growth performance. Furthermore, although it is known that the nutritional status of the egg is sufficient to ensure embryo development until hatching, growth performance during and after hatching is further enhanced by in ovo supply of neonatal exogenous nutrients. The in ovo technique consists of injecting exogenous substances inside hatching eggs to improve embryonic development, hatching, and post-hatch parameters. To the best of our knowledge, few studies have explored the effect of in ovo injection of essential oils. The objective of this study was to explore the effects of in ovo injection of different concentrations of rosemary essential oil on embryonic development and hatching parameters in Japanese quail. The findings indicated that the in ovo injection of rosemary essential oil at a low concentration (1 µL/egg) was beneficial for improving hatching compared to 3 µL/egg, which proved to be toxic to the quail embryo. However, it should be noted that both treatments increased the relative weight of chicks at hatching compared to control groups.

**Abstract:**

This study aimed to determine the effects of air sac injection of rosemary essential oil at different concentrations in ovo in quail eggs on hatching rate, relative chick weight at hatching, and embryonic mortality rate. A total of 1060 Japanese quail eggs were divided into four groups: negative control (non-injected), positive control (30 µL sterile distilled water/egg), and two treated groups with 1 and 3 µL oil/egg, respectively. The concentration of 3 µL/egg showed a toxic effect on embryonic development, as revealed by the significantly (*p* = 0.015) higher post-injection mortality rate (18.21%) compared to 1 µL/egg with 8.3%. Furthermore, hatchability was significantly increased (*p* = 0.0001) with 1 µL/egg compared to 3 µL/egg with 69.1% and 44.48%, respectively. No significant difference was observed between the concentration of 1 µL/egg and the control groups (*p* = 0.822). Both l and 3 µL essential oil/egg significantly enhanced (*p* = 0.0001) relative chick weight at hatching by 67.14% and 70.32%, respectively, compared to the control groups. In conclusion, injecting eggs with 1 µL oil/egg showed positive effects both on hatching and relative chick weight. The concentration of 3 µL/egg was revealed to be toxic, with dramatic effects on embryonic survival.

## 1. Introduction

In ovo injection, as a biotechnological approach, consists of administering into an egg exogenous substances during incubation [1]. This technology is widely used in several avian species, including broilers and quails [2,3].

The in ovo approach was first employed for vaccination [4], and numerous studies have proven its effectiveness against both Marek’s and Newcastle diseases [5,6]. Thus, introducing vaccines on the 18th day of incubation strengthens the chick’s immune system both during and after hatching [4].

Subsequently, further studies have shown interest in injecting other exogenous substances, including prebiotics and synbiotics [7], hormones [8], carbohydrates [9], and plant extracts [10,11] to improve embryonic development, hatching parameters, growth performance, and immune responses. Regarding the improvement of poultry production, interest in phytobiotics, including a wide range of substances, and the effectiveness of their antibacterial properties, have been well documented, with a particular emphasis on essential oils (EOs) [12].

EOs known as plant secondary metabolites have generated great attention in particular due to their various biological properties: antibacterial, antioxidant, and growth stimulating [13]. Therefore, these phytobiotic substances have been widely supplemented in animal diets to improve growth performance, to improve animal production performance, and to enhance livestock productivity [14,15]. Most of these active secondary plant metabolites act as suitable alternative feed additives to improve digestibility and nutrient absorption rather than synthetic antibiotics, which were widely used until recently in the poultry industry [16].

Indeed, the continuous use of antibiotics as growth promoters, even at low concentrations, has led to increased pathogenic resistance and accumulation and transmission of antibiotic resistance genes (ARGs) in animal microbiota to humans and the environment. This has led the EU to ban synthetic antibiotic drugs as growth promoters since 2006 [16,17].

Consequently, and to face constraints related to antibiotic resistance, the poultry industry has highlighted the need to use a broader spectrum of alternative products. This has led researchers to study other molecules with antibacterial and antiviral properties. From this perspective, several studies have reported that enriching or supplementing poultry diets with EOs was effective for improving production and reducing antibiotic use [14,15].

Specifically, *Rosmarinus officinalis* EO appears to be one of the most interesting and efficient supplements in poultry diets; it has been proved to provide antioxidative (effective against oxidative stress), antimicrobial, and growth stimulator effects in different avian species [18,19]. In the last few years, in ovo technology has not been restricted to the improvement of growth performance or the prevention of avian diseases; instead, this technique has been widely used to fight against antibiotic resistance by administering upstream plant extracts (substitute molecules) in relation to their antioxidant, antimicrobial, and growth enhancer properties on the embryonic development of avian species [11,20].

In this respect, numerous studies have established the beneficial effects of in ovo administration of phytobiotics molecules, such as flavones and virgin oils, on embryonic development and growth performance [21,22].

The Japanese quail is a suitable animal model in avian research because of its easy maintenance, early sexual maturity, and fast growth and development [23]. In particular, in ovo feeding has been reported for improving coturniculture production. In this respect, potential increases in growth-related endocrine activity of various compounds, including estradiol benzoate, estrogen, and corticosterone, during the quail’s embryo development have been well documented [8,24,25]. Furthermore, numerous studies have reported the usefulness of early feeding of the quail’s embryo with several exogenous molecules, such as vitamins, amino acids, and plant extracts [11,26,27]. It has been established that introducing these nutrients into eggs during incubation has been shown to be effective in improving hatching rate, chick hatch weight, genetic background, egg characteristics, productive performance, carcass traits, and certain blood serum characteristics. These beneficial effects have been closely related to the potential antioxidant capacity of these bio-active compounds, particularly those of plant extracts.

However, to the best of our knowledge, there are limited published data about the effect of in ovo administration of essential oils. Indeed, Oladokun et al. [28] and Oladokun et al. [29] have explored the effect of in ovo injection of a commercial solution containing a phytonutrient blend of EOs in broilers. The results reported reduced egg hatching rates but benefits to gut morphometry and caecal microbiota.

Nevertheless, even if EOs have generated great interest for their biological properties, their use is limited because of their insolubility in water. Currently, there is rising interest in encapsulation technology, which has been proven to be an efficient solubilization method to prevent the loss of EOs’ volatile active constituents [30]. In this study, we used polyethylene glycol as a potent solubilizer to optimize the biological effect of rosemary EO in the hatching eggs.

From this perspective, this study aimed to investigate the effects of in ovo injection of pure rosemary essential oil at different concentrations on hatch rates, chick weight at hatching, and embryonic mortality rates in Japanese quail (*Coturnix coturnix japonica*).

## 2. Materials and Methods

### 2.1. Rosemary Essential Oil Extraction

Rosemary (*Rosmarinus officinalis* L.) plant was collected in Northeastern Algeria. The harvested plants were dried in the shade for 10 days before extraction.

Extraction of the essential oil was carried out by hydro-distillation method using a Clevenger’s type apparatus (Clevenger, 1928). Distillations were carried out by boiling aerial plant parts for 3 h, and the essential oil was kept in air-tight sealed vials and stored at 4 °C until further use.

### 2.2. Preparation of Treatments

Extracted rosemary essential oil was first solubilized by the use of polyethylene glycol as an emulsifier agent; this led to a complete dissolution by solid dispersion technique.

The injected solutions were prepared a day before the injection and stored at 4 °C. On the day of injection, these solutions were placed under the standard conditions of injection (temperature 37.7 °C, humidity of 60%).

### 2.3. Eggs Incubation

This study was carried out at the University of Bejaia, Algeria. A total of 1060 fertile fresh Japanese quail eggs (*Coturnix coturnix japonica*) were used. Eggs were numbered, weighed individually, and then divided randomly into four equal experimental groups (53 eggs in each group; five replicates) with a similar average weight of 11.58 ± 0.18 g (mean; SE). The treated eggs were injected with a volume of 30 µL of distilled water containing 1 µL or 3 µL of essential oil. The positive control group was injected with only 30 µL of distilled water (vehicle), and the negative control group remained without injection. Eggs were set in an incubator (Cimuka, CT 180 SH Modal) under standard conditions: 55% humidity, temperature of 37.7 °C, with one rotation every 2 h.

### 2.4. In Ovo Injection Procedures

On the ninth day of incubation, eggs from each egg group, except those of the negative control group (non-injected), were injected with the corresponding treatment according to the method reported by Wilhelms et al. [3]. The procedure was carried out under the standard conditions of incubation (temperature of 37.7 °C, humidity of 60%), with disinfection of the injection site with 70% alcohol. The eggshells were carefully punctured with a sterile 21-gauge needle at a 5 mm depth so that solutions could be delivered into the air chamber. Then, immediately after the injection procedure, the injection site was sealed with sterile liquid paraffin in order to avoid bacterial contamination. Finally, the eggs were reinserted into the incubator under standard conditions (temperature 37.7 °C, humidity of 60%). The eggs of the negative control group were also pulled out from the incubator under the same conditions as the other groups.

### 2.5. Hatching Parameters

Hatching parameters were analyzed for all treatment groups. The non-hatched eggs were opened and the dead embryo stages were determined. The following parameters were assessed according to Nakage et al. [31]:

Hatching (%) = (Number of hatched eggs/Total incubated eggs) × 100,(1)

Relative hatching weight (%) = (Absolute hatched chick weight/Initial egg weight before incubation) × 100,(2)

Total embryo mortality (%) = (Number of unhatched fertile eggs/Number of incubated fertile eggs) × 100,(3)

Embryo post-injection mortality (%) = (Number of dead embryos after the 9th day of incubation/Number of unhatched fertile eggs) × 100.(4)

### 2.6. Ethical Approval

All study procedures and Guidelines for Experimental Animals were approved by the Association Algérienne des Sciences en Expérimentation Animale (58 AASEA: N°45/DGLPAG/DVA/SDA/14).

### 2.7. Statistical Analysis

The normality of all data sets was ascertained by testing residuals by the Kolmogorov–Smirnov test in StatView software, version 4.5. Data sets found to be normal were subjected to one-way ANOVA in the same statistical package with experimental treatments as a factor and the relevant data sets as variables. Values were statistically different when the *p* value was (*p* < 0.05) and expressed as mean standard error, SE. The graphics were designed using GraphPad Prism software, version 8.3.0.

## 3. Results

### 3.1. Hatchability

**Figure 1:**
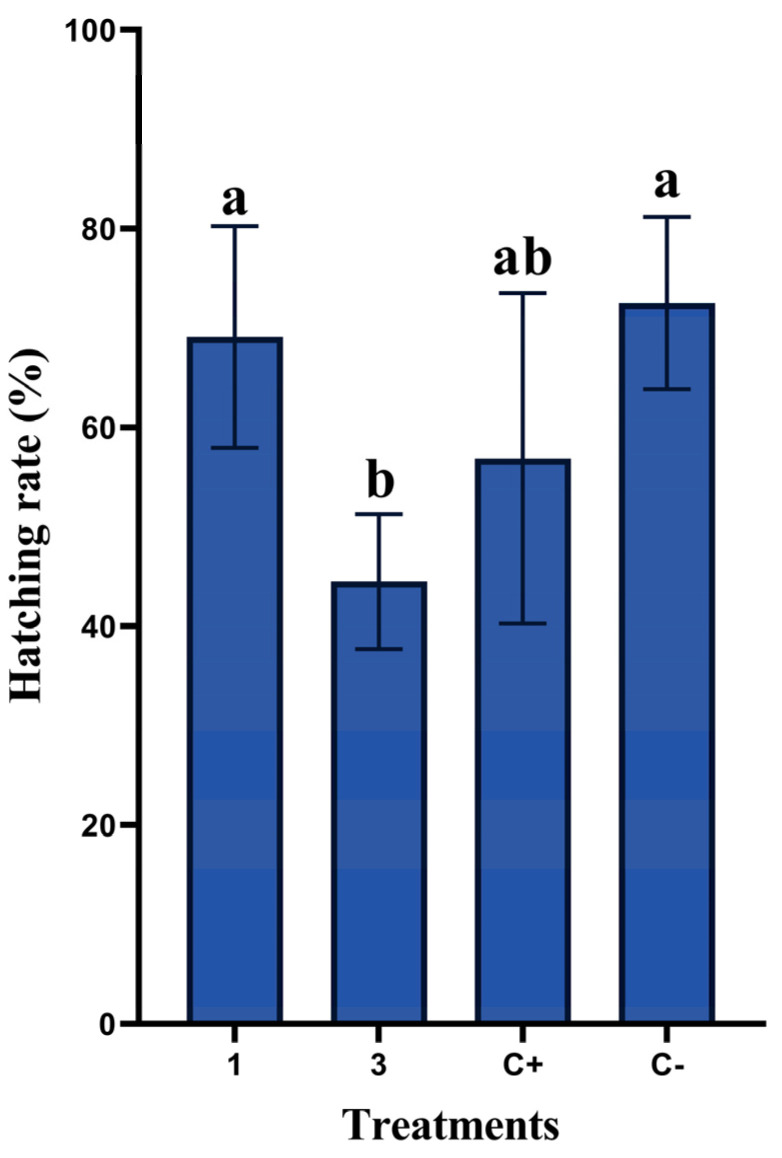
Effects of in ovo administration of different concentrations of rosemary EO on hatchability rates in non-injected eggs (C−), positive control injected with distilled water (C+), and eggs injected with rosemary EO at 1 or 3 µL/egg (five replicates/group). Different letters (a, b) indicate a statistically significant difference (*p* < 0.05). Values are represented as mean ± standard error of the mean (SE).

The effects of the in ovo injection of rosemary essential oil (EO) on hatching rates are presented in Figure 1. The results show that treatment with 3 µL/egg (EO3) of pure essential oil induced a significant decrease (*p* = 0.002) in hatching rates (44.48%) when compared to treatment with 1 µL/egg (EO1) (69.1%). No significant difference (*p* = 0.822) was observed between the non-injected (C−) and EO1 groups. Treatment with distilled water reduced hatching rates to 56.9%, which was not significantly different from C− or EO3 groups (*p* = 0.099).

### 3.2. Total Embryo Mortalities

**Figure 2:**
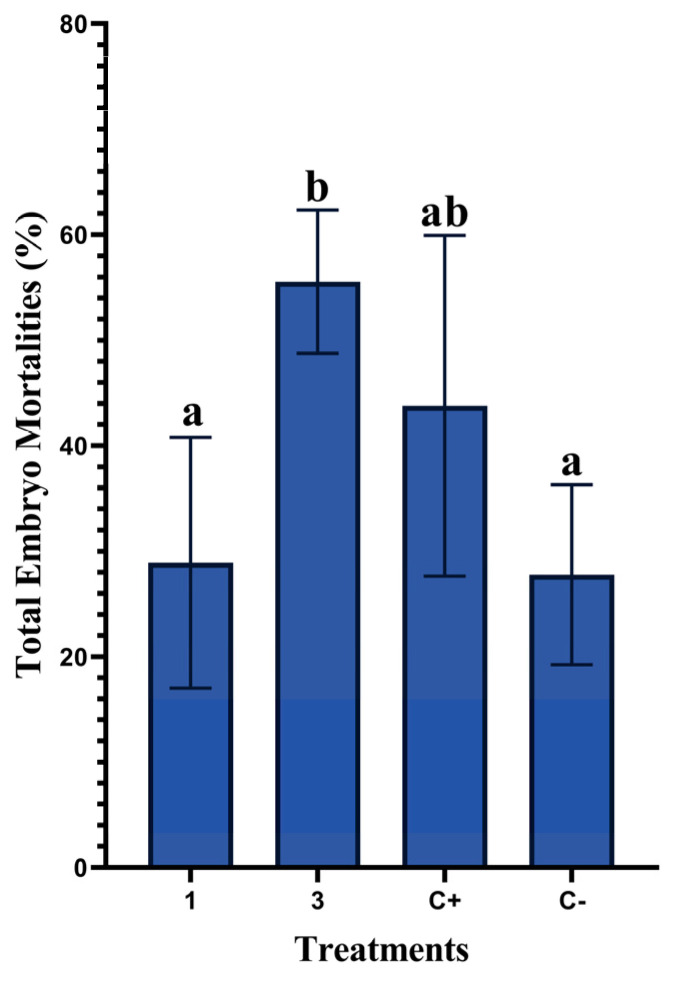
Effects of in ovo administration of different concentrations of rosemary EO on total mortality rates in the non-injected group (C−), distilled-water-injected group (C+), and treatments with rosemary EO at 1 or 3 µL/egg (five replicates/group). Different letters (a, b) indicate a statistically significant difference (*p* < 0.05). Values are represented as mean ± standard error of the mean (SE).

The effects of the in ovo administration of different concentrations of rosemary EO on total embryo mortality rates are summarized in Figure 2. According to the results, significantly higher values (*p* = 0.001) were observed in the 3 µL/egg group compared to the 1 µL/egg group, with 55.53% and 28.89%, respectively. Injecting only distilled water induced high embryonic mortalities (43.77%) compared to the non-injected group (27.77%).

### 3.3. Post-Injection Embryo Mortalities

**Figure 3:**
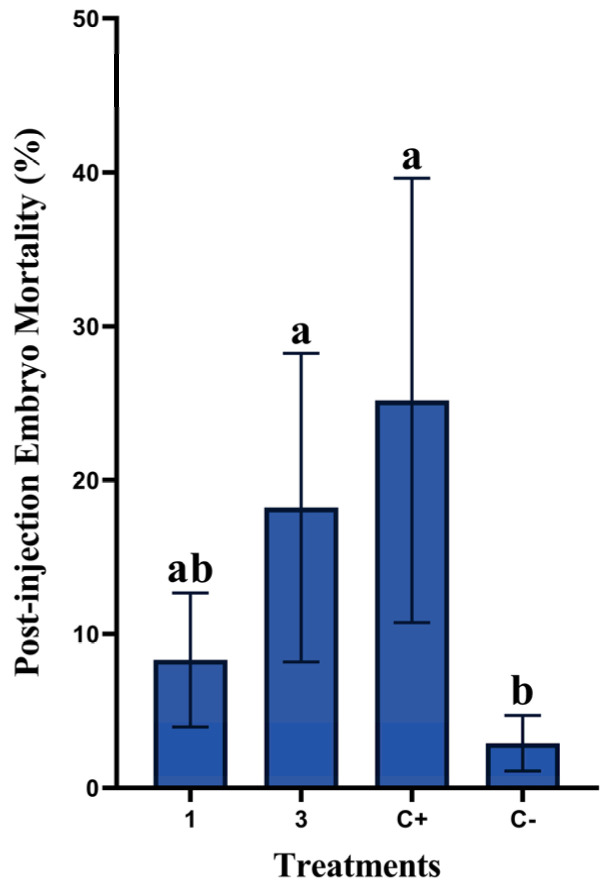
Effects of in ovo administration of different concentrations of rosemary essential oil on post-injection embryo mortality rates in non-injected group (C−), positive control (eggs injected with distilled water) (C+), and in treatments with rosemary EO at 1 or 3 µL/egg (five replicates/group). Different letters (a, b) indicate a statistically significant difference (*p* < 0.05). Values are represented as mean ± standard error of the mean (SE).

Figure 3 summarizes the impacts of the in ovo injection of rosemary essential oil on post-injection embryo mortalities. Injecting 3 µL/egg significantly increased post-injection embryo mortality (18.21%) (*p* = 0.015) in comparison with 1 µL/egg (8.32%).

Furthermore, the injection of distilled water negatively affected embryo survival (embryonic mortality 25.18%).

### 3.4. Relative Weight of Chicks at Hatch

**Figure 4:**
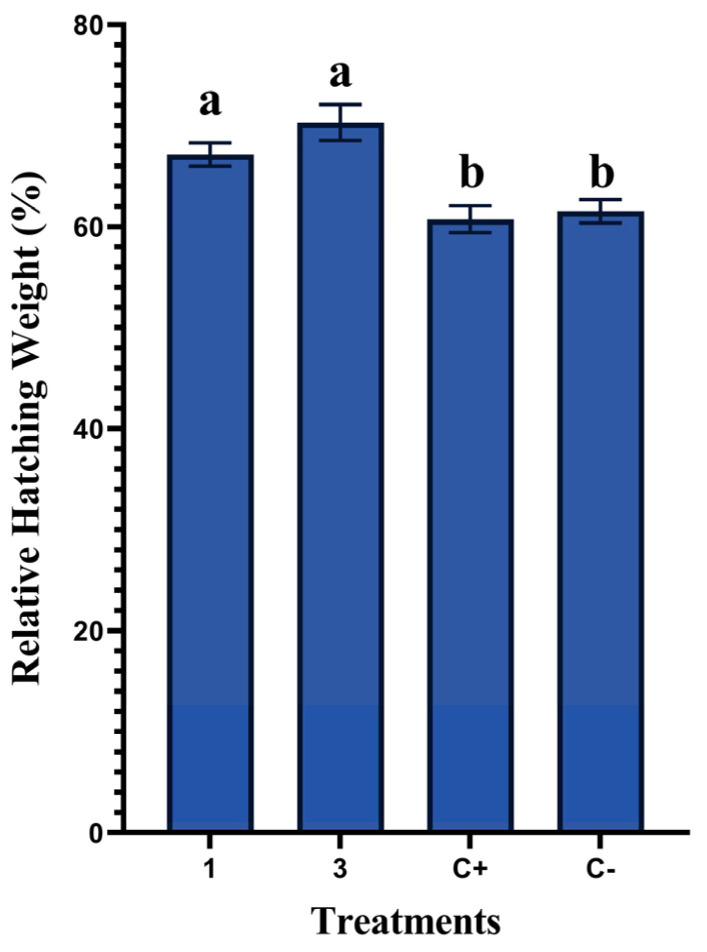
Effects of in ovo administration of different concentrations of rosemary essential oil on relative hatching weight in non-injected group (C−), positive control (eggs injected with distilled water) (C+), and in treatments with rosemary EO at 1 or 3 µL/egg (five replicates/group). Different letters (a, b) indicate a statistically significant difference (*p* < 0.05). Values are represented as mean ± standard error of the mean (SE).

The effects of in ovo injection of rosemary essential oil on the relative weight of chicks at hatch are shown in Figure 4. When compared to the control groups, a statistically significant effect was observed in treatments with EOs (*p* = 0.0001). No significant differences were observed between the treatment groups of 1 µL/egg and 3 µL/egg (*p* = 0.091) or between the control groups (C− and C+) (*p* = 0.696). Treatment with EO3 showed the highest result by recording 70.32% compared to 67.15% in the EO1 group. The non-injected group (C−) and the distilled-water-injected group (C+) showed 61.54% and 61.83%, respectively.

## 4. Discussion

Limited studies have been dedicated to in ovo administration of plant extracts, particularly the sensibility of embryos to essential oils (EOs). In this regard, this study highlights the beneficial and potentially harmful effects of different concentrations of rosemary essential oil, particularly on embryonic mortality, hatching parameters, and relative weight of chicks at hatch. Two treatment groups were considered with 1 µL/egg and 3 µL/egg of pure *R. officinalis* essential oil.

The results of this study revealed that in ovo injection of rosemary essential oil at a low dose of 1 µL/egg significantly increases hatchability (*p* = 0.0001) compared to treatment with a high dose of 3 µL/egg. The value remained similar and without significant differences (*p* = 0.822) compared to that recorded in the negative control group (non-injected). However, hatchability in the positive control (group injected with only distilled water) was lower than that recorded in the non-injected group (Figure 1). This reflects that injecting eggs increases their fragility, but it seems that injecting 1 µL/egg of rosemary essential oil protects the quail embryo and consequently increases chick hatch weight. In this respect, we hypothesize that the positive effect of *Rosmarinus officinalis* EO could be attributed to the wide spectrum of its powerful antioxidant and antimicrobial activities. The improvement of antioxidant capacity during embryonic development is probably the main cause for the enhanced body weight gain. Consequently, other essential oils that have strong antioxidant activity may also result in increased weight gain. In this respect, interest in using rosemary essential oil at low doses to protect animals’ cells has been well documented in the literature [32,33]. The results of this study are in agreement with in vitro studies in ram sperm cryopreservation showing a positive enhancement in spermatozoa mobility and viability [32]. Furthermore, Touazi et al. [33] have reported that male gametes of broiler chickens are best preserved at low concentrations of rosemary essential oils.

On the other hand, the results of this study were not in agreement with those of Oladokun et al. [28], who found that in ovo injection of a commercial solution containing a mixture of essential oils in the broiler caused 18.1% and 19.5% reduction in hatchability compared to the control groups. However, the in ovo inclusion of rosemary oil significantly improved hatchability, as revealed by Sulaiman and Tayeb [22], who investigated the effect of rosemary oil on broiler performance. Administering different levels of rosemary virgin oil has been shown to positively affect broilers’ egg hatching rates [34].

Furthermore, the effects of some plants extracts on broiler chicken growth performance have been reported by Toosi et al. [35], who found that in ovo injection and the inclusion of a blend of essential oils and organic acids (Biacid™) significantly improved the broilers’ hatching eggs.

On the other hand, the injection of natural rosemary oil, known for being less toxic than essential oil, provided a beneficial effect on chicks’ immune system [34]. Similarly, Farag et al. [36] have reported the expression of antioxidant and neuroprotector effects on chick embryos by in ovo injection of Rosmarinic Acid (C_18_H_16_O_8_). According to the authors, rosmarinic acid could be effective against toxic molecules during the embryonic development of broiler chickens. In vivo, Christopoulou et al. [37] and Zhang et al. [38] established that low doses of essential oils in water and feeding additive, especially *Rosmarinus officinalis*, do not present toxicological effects.

Regarding this study, the in ovo administration of rosemary EO at a concentration of 3 µL/egg significantly (*p* = 0.0021) reduced hatchability by recording the lowest rate compared to the other studied groups (Figure 1). 

Furthermore, the significant post-injection embryonic mortalities (Figure 3) clearly demonstrate the toxic effect of this concentration, causing reduced hatchability. However, both 1 µL/egg and 3 µL/egg concentrations reduced post-injection embryonic mortalities when compared to the positive control injected only with distilled water. In this context, Oladokun et al. [28] have also reported in broilers a reduced hatchability when injecting in ovo 100 µL of a commercial solution containing a blend of essential oils.

EOs have been widely used for their various antioxidant and antimicrobial properties, but their adverse effects have also been extensively documented. The current findings are in agreement with several investigations reporting the toxic effects of essential oils in animal models [39,40]. Regarding rosemary EO, it has been established that ingestion of rosemary essential oil at high doses may induce a toxic effect on male rat fertility [41]. Moreover, it has been reported that ingestion of high doses of rosemary extracts or EO may cause abortive effects, gastrointestinal irritation, kidney damage, and neurological effects [42]. Extracted individual molecules from EOs, such as thymol and carvacrol, have also been reported to present toxic effects on avian embryos, even at low concentrations [38]. Furthermore, the administration of aqueous rosemary extract without EO proved to be toxic through the inhibition of embryonic implantation [43]. Indeed, in ovo administration of plant extract has also been reported to have dramatic effects on embryonic development [44]. In addition, Bakkali et al. [40] reported that essential oils have been shown to damage the cellular membrane structure as well as the cytoplasm and cell organelle membranes because of the great number of constituents.

Similarly, thymol and carvacrol as phenolic compounds (considered the most important constituents of essential oils) have been shown to be toxic to the intestinal cells due to their lipophilic and hydrophobic characteristics, which suggests an interaction with the cytoplasmic membrane by affecting its permeability. As a result, the loss of ionic gradients leads to the alteration of essential cellular processes, resulting in the death of the cell [45].

On the other hand, the results of this study revealed that in ovo administration of rosemary essential oil on the ninth day of incubation significantly increased (*p* = 0.0001) the chicks’ hatch weight. However, these results contrast with those reported by Oladokun et al. [28], who noted that no effect was recorded on chick weight when injecting a commercial essential oils blend. This is probably due to the high doses used but also the types of injected products, as in this study, we injected pure essential oil. Interestingly, even the toxic effects expressed by the 3 µL/egg concentration on hatchability and embryonic mortality were associated with enhanced chick weight, probably due to the fact that only resistant chicks could achieve the hatching stage. Moreover, in ovo inclusion of rosemary oil improved the birds’ body weight at hatching, as revealed by Sulaiman and Tayeb [22], who investigated the effect of rosemary oil on broiler performance. The effectiveness of administering different levels of rosemary virgin oil in the improvement of broilers’ body weight at hatching was also proved by Sulaiman and Tayeb [34]. In addition, based on findings reported by Sarhan et al. [10], the in ovo injection of plant extracts, especially clove extract on day 10 of incubation, has shown significant positive effects on broiler hatch weight and post-hatch performance, as well as serum lipid profile and antioxidant activity, compared to the control group.

Furthermore, the effects of encapsulated thyme oil on broiler chicken growth performance have been reported by Yaseen et al. [46], who found that in ovo injection and dietary inclusion of nano-encapsulated thyme oil significantly improved the birds’ body weight and body weight gain of broiler chickens.

On the other hand, Yesilbag et al. [47] reported that supplementing broiler diets with Rosemary essential oil improves body weight gain.

In this regard, it has been shown that the in ovo injection of rosmarinic acid improved the antioxidant status of the chicken embryo [36]. In this respect, Lemonica et al. [43] have reported that rosmarinic acid has one of the most potent antioxidant activities, especially in aqueous and lipidic environments. Eggs being essentially a lipid environment, an underplayed protection mechanism could be due to the solubilization of essential oil active molecules in these conditions. This confirms our hypothesis that the strong antioxidant property of rosemary essential oil played an important role in increasing the chicks’ hatch weight by limiting lipid peroxidation and membrane damage during embryo development. In this context, it has been proven that the beneficial effects resulting from injecting plant extracts in ovo are closely related to the antioxidant capacity of their phenolic compounds, which are known for their ability to trap and neutralize free radicals in hatching eggs during embryo development [11,36,48].

Furthermore, the benefits of using rosemary EO to supplement avian drinking water and diet have been closely related to its potent antioxidant, antimicrobial, and growth promoter properties [19,49].

## 5. Conclusions

On the basis of this experiment, we postulated that pure essential oils at low concentrations might improve embryonic development and hatchability in quail. The results revealed the usefulness of in ovo injection of rosemary essential oil at a lower concentration for better hatchability and higher hatch weight. Toxic effects were observed, as described above, but compared to the injection of distilled water, the findings also revealed that injecting rosemary essential oil prevented damage caused by the eggshell piercing. The results revealed that the enrichment of eggs with rosemary essential oil could be a suitable alternative as a growth promoter in poultry farming. However, further studies are needed to understand which active compounds of rosemary essential oil are responsible for the beneficial and harmful effects observed in this study. In addition, it would be interesting to assess the effectiveness of low concentrations, especially 1 µL/egg, on blood parameters, antioxidant capacity, and post-hatch growth performance.

## Data Availability

All data generated or analysed during this study are reported in this published article. The data sets used and/or analysed in this study are available from the corresponding author upon request.
